# The impact of the COVID-19 pandemic on ICU clinical trials: a description of one research team’s experience

**DOI:** 10.1186/s13063-023-07355-4

**Published:** 2023-05-11

**Authors:** Linda L. Chlan, Mary Fran Tracy, Jessica Ask, Amos Lal, Jay Mandrekar

**Affiliations:** 1 Department of Nursing, Division of Nursing Research, Mayo Clinic College of Medicine & Science, Rochester, USA; 2 School of Nursing, University of Minnesota, Minneapolis, USA; 3 Mayo Clinic, Rochester, USA; 4 Department of Medicine, Division of Pulmonary and Critical Care Medicine, Mayo Clinic College of Medicine and Science, Rochester, USA; 5 Division of Quantitative Health Sciences, Mayo Clinic College of Medicine, Rochester, USA

**Keywords:** Clinical trial, Pandemic, Accrual, Intensive care unit, Critical care

## Abstract

**Background:**

Disruptions to clinical trials conducted in the intensive care unit (ICU) due to the severe acute respiratory syndrome coronavirus 2 (SARS-CoV2; coronavirus disease 2019 [COVID-19]) pandemic included fewer new trials activated and more trials stopped. While a number of ongoing, non-COVID-19 clinical trials remained open to enrollment, the direct impact of the pandemic on ICUs instilled chaos in this already challenging environment. The numerous challenges need to be reported so investigators can proactively plan and manage these myriad challenges. Thus, the purpose of this study was to describe the impact of the COVID-19 pandemic on screening and accrual for a non-COVID-19 parent clinical trial enrolling critically ill ICU patients receiving mechanical ventilatory support.

**Methods:**

A descriptive, retrospective design using quantitative data from detailed screening logs and qualitative observations with field notes from a parent clinical trial were used to address the objectives. The primary aims of the two-site parent clinical trial (*n* = 190) are to test the efficacy of self-administration of sedative therapy by mechanically ventilated ICU patients on anxiety and delirium occurrence. ICUs from two academic medical centers [names removed for blinding] plus a community hospital in Minnesota were screened daily for alert patients (Richmond Agitation Sedation Scale [RASS] − 2 to + 1), following commands, hemodynamically stable with sufficient hand grip strength to depress a push-button device. Screening data were summarized based on the primary reason patients were not enrolled (screen failures, declinations of consent). Descriptive statistics (frequencies, percentages), chi-square, and Fisher’s Exact test were used to describe the data and to determine any differences among distributions of screening failures and recruitment declinations during the defined pre-pandemic (August 27, 2018–March 15, 2020, 2976 screened patients) and pandemic timeframes (March 16, 2020–February 28, 2022, 3912 screened patients). Qualitative data from varied sources such as screening logs, institutional email communications, staff field notes, and research team meeting minutes were summarized into themes.

**Results:**

Despite significantly fewer screen failures due to hypotension, cognitive impairment/dementia, coma, or chemical paralysis with 938 additional patients on the screening log, more were accrued pre-pandemic (*n* = 55) than during the pandemic period (*n* = 45); declination reasons were non-significant. Pandemic burdens experienced by study personnel, ICU care providers, and patients/families were revealed that attributed to decreased accrual.

**Conclusions:**

While the parent clinical trial remained opened, cumulative factors adversely impacted the trial during the pandemic period with fewer patients accrued. The human toll of the pandemic on research staff, clinicians, and patients/family members demands that investigators be proactive in managing these challenges to conduct ICU clinical trials successfully, including careful oversight of human and financial resources.

**Trial registration:**

ClinicalTrials.gov NCT#02,819,141 Registered 29 June 2016.

## Background

Participant enrollment and retention for any research study can be challenging. Coupled with the unpredictable nature of intensive care units (ICU) and tenuous patient status, accrual into ICU clinical trials can be daunting.

A new obstacle for ICU clinical trial patient accrual emerged in March 2020. The severe acute respiratory syndrome coronavirus 2 (SARS-CoV2; coronavirus disease 2019 [COVID-19]) created havoc in China as early as December 2019. COVID-19 was declared a global pandemic on March 11, 2020 [1]. Clinical trials were suspended, terminated, or withdrawn due to the ramifications of the pandemic [2]. Since the early days of the pandemic, COVID-19 has pushed ICU census to unprecedented levels, with severely ill patients requiring prolonged ICU stays, delaying turnover of beds limiting the pool from which to recruit subjects into non-COVID-19 clinical trials [3, 4].

The COVID-19 pandemic adversely impacted clinical research around the globe, regardless of recruitment settings. Some investigators conducting clinical trials outside of hospitals during the early phase of the pandemic were able to pivot to digital platforms with minimal impact to overall accrual [5, 6] Investigators performed systematic reviews of trial registration databases (i.e., clinicaltrials.gov) to describe the impact of the COVID-19 pandemic on activation of new clinical trials, registration of new clinical trials, stoppage of ongoing clinical trials, and completion of clinical trials. Early in the pandemic, there was an estimated 5.8% decrease in the number of clinical trials registered by 219 countries through mid-2020 [7]. Similarly, from March 2019 through 2020, the number of new clinical trials activated in the early pandemic period decreased as did the number of active clinical trials registered in CLINICALTRIALS.gov with an increase in the number of trials stopped [8]. Likewise, the number of non-COVID-19 clinical trials initiated decreased from 2019 to 2021 with a slightly larger decrease in the USA compared to Europe [9]. Persons with impaired immune function, such as those with certain types of cancer, at high-risk for adverse outcomes if they contracted COVID-19 were reluctant to even seek healthcare, thus reducing the number of potential subjects for clinical trials recruited through clinic settings [10]. Unfortunately, pivoting to digital clinical trials for inpatient research studies was impossible. Notably absent from the scientific literature is a description and quantification of the impact of the global pandemic on non-COVID-19 clinical trials conducted in the setting of the intensive care unit. Specifically, little is known about how the COVID-19 pandemic has specifically influenced ongoing ICU clinical research. Thus, the purpose of this study was to describe the impact of the pandemic on one investigative team’s non-COVID-19 parent clinical trial enrolling critically ill ICU patients receiving mechanical ventilatory support.

## Methods and materials

### Overview of parent clinical trial

The primary aims of the two-site, two-group randomized parent clinical trial (R01HL130881) are to test the efficacy of mechanically ventilated patients’ self-administration of dexmedetomidine (*n* = 190) to reduce anxiety, delirium, ventilator days, and ICU length of stay. Patients *must* follow simple commands, be able to use a medication push-button infusion device, and be willing to self-administer a sedative agent to be eligible for enrollment via own informed consent or from a legally authorized representative (LAR). A detailed description of the parent clinical trial and its protocol can be found elsewhere [11]. All research was performed in accordance with human subjects in research guidelines put forth by Mayo Clinic’s Institutional Review Board (IRB) and the University of Minnesota IRB for greater than minimal risk clinical trials. Thus, patient safety is of the utmost importance for enrollment into this highly selective and controlled efficacy clinical trial which yields approximately 25% accrual of all eligible mechanically ventilated patients.

### Impact of the COVID-19 pandemic on the parent clinical trial screening and accrual

A descriptive, retrospective design using quantitative data obtained from screening logs describes the impact of the pandemic on screening and accrual. Research team qualitative observations and field notes illuminate the quantitative results.

Intensive care units from two academic medical centers in Minnesota participated in the parent clinical trial. The first site recruited patients from a 2059-bed academic medical center with 161 adult/pediatric/neonatal ICU beds. Enrollment occurred in a sub-set of 80 ICU beds across Medical, Surgical, Trauma, and Neurological ICUs. The second site recruited patients from two hospitals. The first hospital is an 850-bed academic medical center with 62 ICU beds across Medical, Surgical/Neuro, and Cardiovascular ICUs. The second hospital is a 390-bed academically-affiliated community medical center with a 25-bed mixed medical/surgical/cardiovascular ICU.

A 3-step screening process prior to informed consent consists of (1) electronic health record (EHR)-automated reports of mechanically ventilated patients, (2) in-depth EHR review for inclusion criteria, then (3) bedside assessment of grip strength to use a push-button medication delivery device, mental status/alertness assessment, and ability to follow commands. Mechanically ventilated patients who are positive for COVID-19 disease are not enrolled in order to preserve personal protective equipment (PPE) for bedside staff and reduce the risk of infectious exposure to research staff.

### A retrospective review of COVID-19 in the State of Minnesota

The first confirmed COVID-19 case in Minnesota (Midwestern United States) was on March 6, 2022. Minnesota saw a rise in COVID-19 cases in early March 2020, reaching the first peak in October of 2020 [12]. Most non-COVID-19 research studies were suspended immediately due to rapidly changing institutional policies, public health policies, and emergency needs to address staffing across inpatient and outpatient settings, inclusive of pop-up swab testing clinics. Large multinational survey-based research from 127 centers showed that decisions to temporarily halt clinical research in non-COVID-19 clinical trials during the initial phase of the pandemic varied widely [13]. Our experience was no different.

As a result of these shifts in personnel and institution-mandated research priorities, the two-site parent ICU clinical trial was first paused on March 16, 2020. Initial re-activation of the parent study occurred at the first site in June 2020; the second site was reactivated in September 2020. Over the next 2 years, prospective enrollment into this clinical trial morphed into “start-stop” cycles due to peaks in ICU admissions with COVID-19-positive patients receiving prolonged ventilatory support. The changing structure of SARS-CoV-2 gave rise to two subsequent major peaks after the initial insult. The delta surge occurred in Minnesota from July 2021 to November 2021; the omicron surge lasted from December 2021 until February 2022. With every variant surge, there were concordant increases in ICU admissions throughout Minnesota. Compounding high ICU census were patients requiring lengthy periods of mechanical ventilatory support leading to longer than average ICU stays. Furthermore, there was an increase in the number of staff who became ill, leading to staff shortages. Lastly, a temporary pause on elective surgeries at the two participating sites led to decreases in eligible post-operative patients, contributing to a severely limited pool of subjects to screen for enrollment. The dynamic nature of the COVID-19 pandemic and its impact on healthcare was profound yet largely not reported in the critical care literature.

### Variables and their measurement

The daily screening and enrollment logs were the primary data source. The pre-pandemic timeframe was August 27, 2018, to March 15, 2020. The pandemic timeframe was from March 16, 2020, to February 28, 2022. These timeframes were selected to reflect both participating sites being fully operational in all aspects of the clinical trial and capture the pandemic timeframe, including significant variant surges.

Data from the screening logs were abstracted and summarized by numerical codes for the primary reason why mechanically ventilated patients were not eligible for enrollment (i.e., screen failures). Data were summarized as percentages based on the primary code combined for both participating sites and by each site, respectively.

Similarly, data were summarized by a percentage of patients’ eligible but declined enrollment. These data are presented combined across sites and each site separately. Accrual data are the number of patients with informed consent and enrolled on protocol pre-pandemic and during the pandemic timeframe.

Qualitative data were abstracted from varied sources including screening logs, email notifications from the institutions and their respective IRBs, study personnel emails, staff field notes, and minutes from weekly and biweekly research team meetings during the pandemic timeframe. These data were summarized into themes that illuminate the pandemic’s impact on screening and enrollment from the perspective of site research staff.

### Analysis plan

Descriptive statistics (frequencies and percentages), chi-square, and Fisher’s Exact test were used to determine differences among distributions of screening failures and why eligible patients declined study enrollment from the defined pre-pandemic and pandemic timeframes with both participating sites combined as well as each site separately.

Data of observations and notes from research study personnel during the pandemic timeframe was summarized into themes by two authors (MFT, JA). Study staff consisted of several study coordinators who screened EHR up to three times per day assessing for changes in eligibility. Patients nearing eligibility were followed by the study team and given a numeric code as to why they were currently ineligible; these codes followed the patient until extubation. For example, if a patient was ineligible and coded as having dementia or language barrier, they were no longer followed; if a patient had a weak grip, they were closely followed by study staff. Study staff worked closely to assess for eligible and ineligible features with patients and their health records.

## Results

### ICU patients not eligible for study inclusion

Prior to the pandemic, the most common reasons why mechanically ventilated patients on the participating ICUs were not eligible was due to Richmond Agitation-Sedation Scale (RASS) scores outside of the eligibility range. Other frequently occurring reasons for non-eligibility included hypotension, acute stroke or persistent seizure activity, unable to follow simple commands, or no sedation received in the previous 24 h. During the pandemic, there was no difference in the percentage of mechanically ventilated ICU patients assessed as ineligible due to RASS scores. During the pandemic, there were more non-COVID-19 patients unable to follow simple commands or had an English language barrier. Fewer patients had hypotension, acute stroke/seizure activity, cognitive impairment, dementia, and severe bradycardia, were chemically paralyzed, required extracorporeal membrane oxygenation, or required heavy procedural sedation (Table 1).

**Table 1:** Screen failures prior to COVID-19 and during COVID-19 both sites combined

Reason for screen failure	Frequency prior to COVID *N* = 2976	Frequency during COVID *N* = 3912	*p*-value*
Hypotension outside eligibility parameters	361 (12%)	322 (8%)	< 0.0001*
Paralysis/unable to use push-button device	73 (2.5%)	76 (2%)	0.18
Acute stroke/seizures	243 (8%)	132 (3%)	< 0.0001*
Cognitive impairment	102 (3%)	27 (1%)	< 0.0001*
Coma	23 (1%)	3 (0.1%)	< 0.0001*
Dementia	75 (3%)	47 (1%)	< 0.0001*
RASS outside eligibility parameters	776 (26%)	990 (25%)	0.64
Chronic ventilator support in residence	45 (2%)	47 (1%)	0.3
Language barrier	104 (3%)	184 (5%)	0.01*
Chemical paralysis	60 (2%)	41 (1%)	0.002*
No sedation in previous 24 h	150 (5%)	152 (4%)	0.02*
Unable to follow simple commands	180 (6%)	326 (8%)	0.0002*
Need for multiple sedative medications	43 (1.5%	57 (1.5%)	1.0
ECMO	89 (3%)	81 (2%)	0.02*
Alcohol withdrawal protocol requiring specific sedative medications	92 (3%)	52 (1%)	< 0.0001*
Temporary pacemaker/severe bradycardia	90 (3%)	67 (2%)	0.004*
Procedure(s) requiring heavy sedation	58 (2%)	50 (1%)	0.03*
Documented research opt-out	47 (1.5%)	13 (0.3%)	< 0.0001*
COVID-19 positive	–––	855 (22%)	––

Percentages do not sum to 100%; *p* < .05*

*Abbreviations*: *COVID-19* SARS-CoV-2, *ECMO* extracorporeal membrane oxygenation, *RASS* Richmond Agitation-Sedation Scale

Tables 2 and 3 present screening failures by site prior to and during the pandemic timeframe. The Mayo Clinic site (Table 2) had more screen failures during the pandemic due to RASS scores, with more patients requiring multiple sedatives. Fewer mechanically ventilated patients received heavier procedural sedation, were unable to follow commands, were on an alcohol withdrawal protocol, or had less hypotension, acute stroke/seizures, or coma. At the University of Minnesota site (Table 3), there were fewer patients ineligible due to hypotension, acute stroke/seizures, coma, cognitive impairment, RASS scores outside of range, requiring chemical paralysis or alcohol withdrawal management protocol, severe bradycardia, and fewer documented research opt-outs. During the pandemic period, more screen failures were attributed to English language barrier, unable to follow commands, required multiple sedative agents, or heavier procedural sedation.

**Table 2:** Screen failures prior to COVID-19 and during COVID-19 Mayo Clinic

Reason for screen failure	Frequency prior to COVID *N* = 1502	Frequency during COVID *N* = 2292	*p*-value*
Hypotension outside eligibility parameters	167 (11%)	154 (7%)	< 0.0001*
Paralysis/unable to use push-button device	35 (2.5%)	49 (2%)	0.78
Acute stroke/seizures	83 (6%)	53 (2%)	< 0.0001*
Cognitive impairment	44 (3%	20 (1%)	< 0.0001*
Coma	21 (1.5%)	3 (0.1%)	< 0.0001*
Dementia	36 (2.5%)	45 (2%)	0.4
RASS outside eligibility parameters	402 (27%)	734 (32%)	0.0006*
Chronic ventilator support in residence	32 (2%)	28 (1%)	0.3
Language barrier	60 (4%)	108 (5%)	0.33
Chemical paralysis	22 (2%)	23 (1%)	0.22
No sedation in previous 24 h	127 (9%)	120 (5%)	0.0001*
Unable to follow simple commands	139 (9%)	148 (7%)	0.00017*
Need for multiple sedative medications	33 (2%)	13 (0.6%)	< (0.0001*
ECMO COVID patients in CV (ICU)	2 (0.1%)	0 (0%)	0.16
Alcohol withdrawal protocol requiring specific sedative medications	71 (5%)	45 (2%)	< (0.0001*
Temporary pacemaker/severe bradycardia	0 (0%)	0 (0%)	––
Procedure(s) requiring heavy sedation	56 (4%)	34 (2%)	< (0.0001*
Documented research opt-out	0 (0%)	1 (0%)	––
COVID-19 positive	–––	524 (23%)	––

Percentages do not sum to 100%; *p* < .05*

*Abbreviations*: *COVID-19* SARS-CoV-2, *ECMO* extracorporeal membrane oxygenation, *RASS* Richmond Agitation-Sedation Scale

**Table 3:** Screen failures prior to COVID-19 and during COVID-19 University of Minnesota

Reason for screen failure	Frequency prior to COVID *N* = 1474	Frequency during COVID *N* = 1620	*p*-value*
Hypotension outside eligibility parameters	194 (13%)	168 (10%)	0.02*
Paralysis/unable to use push-button device	38 (3%)	27 (2%)	0.10
Acute stroke/seizures	160 (11%)	79 (5%)	< 0.0001*
Cognitive impairment	58 (4%)	7 (0.5%)	< 0.0001*
Coma	2 (0.1%)	0 (0%)	< 0.0001*
Dementia	39 (3%)	2 (0.1%)	0.23
RASS outside eligibility parameters	374 (25%)	256 (16%)	< 0.0001*
Chronic ventilator support in residence	13 (1%)	19 (1%)	0.5
Language barrier	44 (3%)	76 (5%)	0.01*
Chemical paralysis	38 (3%)	18 (1%)	0.003*
No sedation in previous 24 h	23 (1.5%)	32 (2%)	0.41
Unable to follow simple commands	41 (3%)	178 (11%)	< 0.0001*
Need for multiple sedative medications	10 (0.7%)	44 (3%)	< 0.0001*
ECMO	87 (6%)	81 (5%)	0.34
Alcohol withdrawal protocol requiring specific sedative medications	21 (1.5%)	7 (0.5%)	0.004*
Temporary pacemaker/severe bradycardia	90 (6%)	67 (4%)	0.02*
Procedure(s) requiring heavy sedation	2 (0.1%)	16 (1%)	0.0015*
Documented research opt-out	47 (3%)	12 (0.7%)	< 0.0001*
COVID-19 positive	–––	331 (20%)	––

Note: Percentages do not sum to 100%; *p* < .05*

*Abbreviations*: *COVID-19* SARS-CoV-2, *ECMO* extracorporeal membrane oxygenation, *RASS* Richmond Agitation-Sedation Scale

### Declination of enrollment

For patients eligible for an informed consent discussion, overall, there was no difference in the distributions of reasons for enrollment declinations prior to the pandemic as compared to during the pandemic. Likewise, there were no differences at the Mayo Clinic site. However, there was a significant difference between distributions of consent declination reasons pre-pandemic compared to during the pandemic at the University of Minnesota site. A higher percentage of primary ICU physicians declined to have the patient enrolled, the site study physician did not deem the patient eligible, or there was no LAR available for consent. There were more patients who withdrew consent during the pandemic timeframe. See Table 4 for details.

**Table 4:** Primary reason eligible patients not enrolled prior to COVID-19 and during COVID-19

Reason not enrolled	Frequency prior to COVID	Frequency during COVID
Legally authorized representative declined consent		
Both sites total	27%	16%
Mayo Clinic	14%	17%
University of Minnesota	38%	15%
Patient declined consent		
Both sites total	51%	59%
Mayo Clinic	60%	71%
University of Minnesota	44%	45%
No legally authorized representative available		
Both sites total	7%	4.5%
Mayo Clinic	7%	0%
University of Minnesota	6%	10%
Primary patient care team declined enrollment		
Both sites total	11%	14%
Mayo Clinic	19%	12.5%
University of Minnesota	4%	15%
Study physician declined enrollment		
Both sites total	0%	4.5%
Mayo Clinic	0%	0%
University of Minnesota	0%	10%
Other		
Both sites total	4%	0%
Mayo Clinic	0%	0%
University of Minnesota	8%	0%
Patient withdrew consent after enrollment		
Both sites total	0%	2%
Mayo Clinic	0%	0%
University of Minnesota	0%	5%

Overall differences between distributions Fisher’s exact test both sites total *P* = 0.11

Overall differences between distributions Fisher’s exact test Mayo Clinic *P* = 0.48

Overall Differences between distributions Fisher’s exact test University of Minnesota *P* = 0.03*

### Participants enrolled

**Fig. 1:**
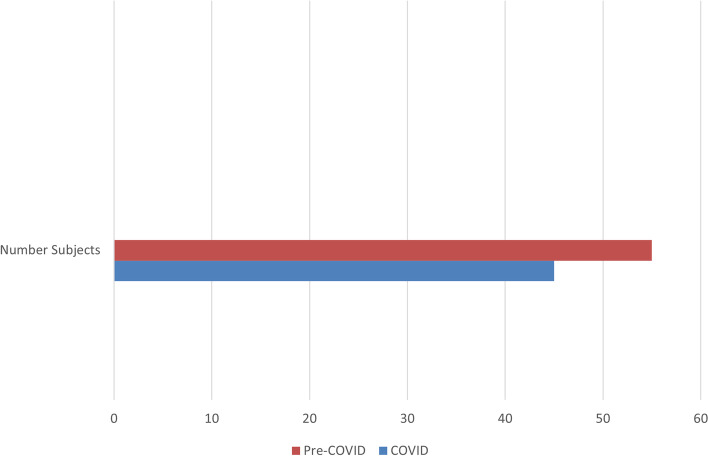
Number of subjects enrolled prior to the COVID-19 pandemic and during the pandemic timeframe

With both participating research sites combined, 22% of mechanically ventilated patients were designated COVID-19 positive, thus not eligible for enrollment. Given that almost a quarter of the ICU census was not eligible is one major reason why fewer patients were enrolled during the pandemic timeframe. See Fig. 1.

### Observations and experiences during pandemic period

#### Mayo Clinic Rochester, MN

Qualitative data recorded by research personnel were summarized into three general themes: study personnel, ICU care providers, and patients/families.

##### Study personnel

Challenges were at an all-time high, given the unrelenting strain on nursing staff and high COVID-19-related patient admissions. All non-patient care departments were asked to assist in COVID-19 relief efforts. Two RN study coordinators were re-allocated to telephone triage for 4 months, with subsequent requests to assist with swab clinics and telephone information lines during the pandemic timeframe. While important to assist burdened front-line staff, this resulted in little to no coverage to screen and enroll research participants. Furthermore, during the pandemic period, the research team experienced several study pauses put in place by institutional leadership as well as by the principal investigator to protect study coordinators’ health by not exposing them to COVID-positive patients.

##### ICU care providers

Once non-COVID-19 clinical research was reactivated, staffing challenge repercussions on the ICUs were evident. ICUs were short-staffed, resulting in multiple nurses caring for patients enrolled in the study in a 24-h timeframe risking the loss of consistency on a high-risk study protocol. One enrolled patient had a travel nurse whose contract was ending at 1100, which necessitated transitioning a study patient to the charge nurse until additional night shift nurses arrived. Nurses expressed frustrations to study coordinators about adding tasks to their already unpredictable shifts.

Physicians were increasingly busy providing care to severely ill ICU patients leading to delays in communication with study coordinators regarding eligible patients. With a short window of study eligibility, these delays risked accrual. Prior to the pandemic, study coordinators would attend ICU rounds. However, this became difficult with changes in day-to-day and ICU processes used to adapt to the high census of severely ill patients.

##### Patients and families

At any given time, almost one quarter of the ICU census contained patients who were COVID-19 positive, leaving very few patients eligible. Once these patients were no longer infectious, given the long periods of high sedative needs, a trend of diminished grip strength with profound hand weakness led to insufficient strength to safely use the medication push-button device. Furthermore, due to high sedative needs, many patients were unarousable and not eligible for participation. Some of these patients were followed by study coordinators for weeks, the profound effects of high sedation, decreased arousability, and weakness took much longer with post COVID patients than past experiences of ICU care.

The visitor policy fluctuated as well during the pandemic timeframe. Visitors were restricted during peaks of delta and omicron surges, leading to difficulty in discussing study details and informed consent with patients’ LARs. LARs could authorize consent via remote, digital signatures but not having families at the bedside severely limited interactions and information exchange. Due to study pauses, visitor restrictions, and a high census of COVID-19-positive patients, otherwise eligible patients were not offered the opportunity to participate.

#### University of Minnesota, Minneapolis, MN

Further complicating matters, institutional leadership from the two participating research sites had different policies during the pandemic period. For example, lengths of time for pauses in non-COVID-19 research activity, access for research personnel to be on-site, and different processes for “re-activating” temporarily suspended clinical research studies contributed additional barriers to screening and accrual. These policies were enacted by consensus between the respective site medical centers and their IRB with ongoing communication to all research teams via email.

##### Study personnel

The pandemic had multiple impacts on study personnel staffing. For example, one RN study coordinator felt compelled to increase work hours as an ICU staff nurse to support pandemic staffing, eventually leading them to leave the study coordinator position. The coordinator stated, “I think it is best for me to leave the PCS study as I feel my role/responsibilities as a bedside/inpatient nurse during COVID-19 takes priority and is too demanding at this time to take on both positions.” Hiring new coordinators was delayed due to temporary university hiring freezes resulting in strict approval processes for new hires, including those supported by research grant funds.

Once the clinical trial was reactivated, onboarding new coordinators were delayed as departments responsible for providing key access and technology permissions were backlogged with managing workloads via new remote processes. For one study coordinator, it took 2.5 months to get the required approvals for the coordinator to be able to function in all components of their role. Orienting new study coordinators was challenging with ongoing mandates that personnel could not be on-site in offices without ongoing special approval. Hospitals were strict about being on-site so introducing new coordinators to ICU staff was not possible. With investigators restricted in being onsite, providing in-person, real-time guidance for coordinators was suboptimal.

##### Patients and families

Keeping study coordinators safe was a top priority for the investigators. The multiple principal investigators elected not to approach COVID-19-positive patients in the early pandemic even if they would have met eligibility criteria out of an abundance of caution. This naturally limited the number of patients eligible for recruiting. As the pandemic continued and more ICU patients with COVID-19 were surviving, eligibility criteria were revised to include patients who had recovered from COVID-19 and were no longer infectious. Coordinators reported in weekly meetings that they were observing issues with patients having adequate strength to be able to participate in the study. Similar to the Mayo Clinic site, this population was extremely weak and did not have sufficient grip strength required to pass pre-screening eligibility criteria.

##### ICU care providers

While ICU team members had been supportive of the study pre-pandemic, there was hesitation to engage with the research during the pandemic. Care providers were overwhelmed with caring for the surge of complex COVID-19-positive patients. ICU staff were reluctant to add any perceived additional work in supporting non-COVID research. In addition, with both nurse and physician turnover throughout the pandemic and the inability of the investigators to be onsite due to medical center policies, it was not feasible to initiate or maintain the in-person, collegial relationships required to foster study conduct in the ICU. Team discussions included reviewing situations where physicians were more hesitant to provide approval to allow enrollment of eligible patients.

## Discussion

The human toll of the global COVID-19 pandemic is immense. In addition to ICU care teams, healthcare workers, and patients/families, the pandemic has impacted the conduct of our parent clinical trial. ICU clinical trials are historically difficult without a pandemic; consenting mechanically ventilated patients in the ICU setting takes skill and confidence from the study team. The impact from the pandemic and the increase in declinations caused staff to feel the pressures of three times a day screening with very few eligible patients to even assess, making an already difficult role of ICU recruitment to feeling nearly impossible. Study staff, including PIs needed to offer extra support to one another, meetings with study team were valued to discuss the successes and challenges all were facing. No one knew how long the pandemic would last, and as time went on with no lifting of restrictions, the moral was challenged. People want to be successful in their roles and with going a month or longer without an enrollment tested the teams’ moral.

Similar to the experiences documented in the literature, our ongoing ICU clinical trial was able to remain open despite institutional policies to pause non-COVID-19 research. The ability for our ICU clinical trial to remain open yet not accruing was only possible due to policy changes from the funding agency. The National Institutes of Health (NIH) leadership quickly realized the necessity to shift national research efforts toward numerous COVID-19 focused investigations. Ongoing clinical trials were directed to communicate regularly with program funding staff to keep them apprised of the ever-changing impact of the pandemic on healthcare institutions that subsequently impacted the ability to conduct patient-centered research. Pausing active screening and enrollment led to decreased grant fund disbursements given new subjects were not enrolled and research staff were reallocated to COVID-19 efforts. This resulted in a cost-savings that allowed clinical trial continuation once the direst phases of the COVID-19 pandemic subsided.

The specific experiences reported in this article created by the global COVID-19 pandemic can be summarized into three broad categories: varying number of ICU patients eligible for trial inclusion; declinations of trial participation by patients, family, and/or ICU medical staff; and clinical trial management and oversight. We outline a number of strategies to mitigate these challenges that might be applicable to clinical trial disruptions from future pandemics.

### Patients eligible for trial enrollment and declination rates

Despite upwards of 936 more mechanically ventilated patients on the screening logs during the pandemic timeframe, we enrolled 10 fewer patients with a trend towards more patient declinations. Overall, patient declinations increased by 8% during the pandemic timeframe, while LAR declinations actually decreased by 11%, perhaps due to fewer patients available for study eligibility. While not statistically significant when both sites’ screen failure data were combined, any increase in patient and LAR declinations significantly impacted accrual in a highly controlled efficacy clinical trial. The Mayo Clinic experienced an increase in both patient and LAR declinations during the pandemic. The reason(s) why significantly more patients were ineligible due to English-language barrier during the pandemic period is uncertain. We do not have any data or observations to explain this result.

When data from both sites were combined, there was no statistically significant difference in terms of RASS scores before versus during the pandemic; the analysis by site showed a larger proportion of patients at the Mayo Clinic had out-of-range RASS scores making them ineligible. Overall, there had been a trend towards deeper sedation for COVID-related acute respiratory distress syndrome (ARDS) patients to avoid ventilator dyssynchrony expecting better outcomes [14, 15]. However, results from a cohort study comparing outcomes based on level of sedation demonstrated that deeper sedation was independently linked to worse outcomes [16]. A similar trend was seen at the University of Minnesota site where a larger, statistically significant proportion of patients required multiple sedative medications during the pandemic phase as compared to the pre-pandemic phase which also affected trial enrollment eligibility (0.7% versus 3.0%, *p* < 0.0001). This also translated into inability to follow commands (3.0% versus 11.0%, *p* < 0.0001), a crucial eligibility criterion to be able to self-administer sedative therapy.

One solution to address fewer eligible patients for screening/enrollment along with more declinations for participation is to increase the number of sites from which to accrue patients. Large institutions may offer additional ICUs that can be added to study protocols, particularly given COVID-19 patients in need of invasive life-support modalities typically were admitted exclusively to specific ICUs. Another strategy is a careful examination of inclusion/exclusion criteria. There may be criteria amenable to revisions to increase the number of eligible ICU patients while maintaining rigor to the study protocol.

The University of Minnesota site had an increase in patients’ withdrawal of consent. While research subjects have the right to withdraw, it is important that investigators ascertain the reasons for this. Investigators should reassure subjects that their standard ICU medical care is unchanged with participation in the clinical trial and that the highly trained ICU nurses are vigilant in their care of all patients. It is quite common for ICU patients to fatigue easily, leading to unwillingness to participate in assessments/data collection that are only part of the study protocol. It may be appropriate to negotiate with a subject to reduce the number of protocol-only assessments or conduct them during the time when nursing staff are already assessing patients. Research staff should focus on collecting any patient response data required for the primary aims on which the power of a clinical trial is based. Obtaining fewer data points is superior to losing all future data collection from enrolled subjects.

The percentage of primary ICU physician and study physician declinations to enroll a mechanically ventilated patient increased during the pandemic. While the reasons for the increase in physician declinations are unclear, several factors could be proposed. During the early phase of the pandemic, there was an uncertainty about “ideal sedation management” of the COVID-19 population, and this uncertainty potentially spilled over to other ICU patients. The optimal approach to sedation in COVID-19 has remained uncertain; however, recent literature is in favor of adhering to available best practices [17, 18]. Another potential enrollment barrier during the pandemic could be that bedside clinicians did not want another factor that could go wrong in an already complex situation, in addition to the existing stress from realignment and lack of resources [19]. A similar sentiment was reported by patients with cancer and their families as the literature conveyed a significant reluctance to participate in ongoing or new clinical trials despite a good track record in conducting clinical trials in academic and research centers [20]. There are times during the conduct of a multi-year clinical trial that maintaining excellent relationships with clinical staff are more important than subject enrollment. If necessary, principal investigators can engage clinical staff to probe further on the reason(s) for enrollment declinations. There may be misunderstandings that can easily be resolved through amicable, professional conversations.

One action taken was to implement electronic consents in order to optimize consent discussions and answer questions when LARs were not physically available on site. Physician investigators become more involved in having direct conversations with provider teams to explain study requirements and alleviate concerns as feasible rather than relying primarily on study coordinator conversations with providers. In addition, each site created a brochure that could be distributed to patients, families, and care providers as a means to convey concise study information that could be referenced in the absence of team member onsite presence. One site initiated weekly research team meetings to discuss trends, monitor and address ongoing policy/procedure changes, and problem solve issues. In addition, this research team-initiated use of a secure texting app in order to have timely remote conversations that could involve all team members in problem solving, rather than 1:1 phone conversations that limited the number of team members in the discussion. Meanwhile, the weekly meetings between sites were even more important to understand the differing approaches taken by the institutions while trying to maintain consistency as feasible with protocol implementation.

The previously mentioned brochures and conversations were important in supporting collegial and respectful conversations about participant eligibility, enrollment, and ongoing status while on study.

### Clinical trial management and oversight

The challenges observed were similar across study sites, yet each site also had unique experiences. The differing institutional approaches to managing research priorities and onsite COVID-19 ICU policies (e.g., visiting policies, ability for onsite work, redeployment of or open coordinator positions, etc.) required even more purposeful communication and coordination between sites.

It is imperative that investigators manage resources appropriately, both human and financial. The numerous start-stop cycles to our clinical trial over the course of the pandemic have negatively impacted morale as well as accrual. Investigators need to monitor the physical and psychological health of research staff to ensure all members continue to function in an effective manner, particularly when patients and/or LARs decline study enrollment. Research staff need to be supported by investigators by recognizing their efforts to enroll critically ill patients into clinical trials. Workloads should be monitored closely. Debriefing sessions to discuss reasons for screen failures or participation declinations need to be conducted in an open, non-judgmental manner. Goodwill efforts such as refreshments, food, or other tokens of appreciation (e.g., handwritten notes of thanks) contribute to a positive work environment that will motivate all members of the research team to excel.

Financial resources need to be closely tracked during periods of enrollment pauses as well as when screening resumes. As evidenced at the University of Minnesota, finances and hiring practices were not only dependent upon study needs and grant funds but also on the University’s overall policies related to budget management and hiring of study personnel. Managing the budget was imperative, though some aspects of financial decisions were outside the researchers’ purview during this extraordinary time such as mandated salary reductions.

#### Limitations

The first limitation of this study is that the pre-COVID and COVID-19 comparison time periods are not precisely equivalent. Likewise, institutional policies during the COVID-19 comparison period were not similar between our two participating centers and may not be similar to other hospitals across the USA. The data source for this descriptive study was screening logs which were not subject to data cleaning leading to possible errors in these data. Finally, the data and experiences reported may be unique to Minnesota and are not generalizable to other ICU clinical trials around the world.

We have found that patients, family members, and ICU clinicians have typically been willing to participate in research initiatives in our settings. However, the prolonged chaos of the pandemic seemed to create such uncertainty, leading to increased hesitancy to support or participate in anything other than the treatments necessary for care. In-depth qualitative investigations are warranted to fully describe the clinical care team and patient/family perspectives on prospective enrollment in ICU clinical trials, along with identification of potential recruitment barriers yet to be uncovered with suggestions for strategies to overcome these impediments to accrual.

#### Summary and conclusions

Each new SARS-CoV-2 variant resulting in a surge of cases can potentially impact ICU admissions and, thus, accrual into any ICU clinical trial. Proactive management and oversight of all aspects of a clinical trial are needed given the new virus variants emerging. Despite the parent clinical trial being focused on “optimizing” sedative therapy based on individual patient needs, a fair degree of hesitancy for study participation as well as heterogeneity in clinical practice patterns have been encountered. Principal investigators are encouraged to remain vigilant in order to maintain productivity in attaining accrual milestones while preserving finances as well as the health and well-being of the entire research team.

## Data Availability

The datasets generated and/or analyzed during the current study are currently not publicly available until the completion of the parent clinical trial but may be available from the corresponding author on reasonable request.

## Abbreviations

ICU: Intensive care unit
SARS-CoV2: Severe acute respiratory syndrome coronavirus 2
COVID-19: Coronavirus disease 2019
RASS: Richmond Agitation Sedation Scale
LAR: Legally Authorized Representative
EHR: Electronic Health Record
RN: Registered nurse
ARDS: Acute respiratory distress syndrome
